# The analysis of telephone consultation contents of patients with bipolar disorder received by a self‐help group

**DOI:** 10.1002/pcn5.20

**Published:** 2022-06-23

**Authors:** Yuko Isogaya, Chiho Suzuki, Shingo Hoshina, Masashi Nibuya, Eiji Suzuki

**Affiliations:** ^1^ Department of Psychiatry Tohoku Medical and Pharmaceutical University Hospital Sendai Japan; ^2^ The Japanese Alliance of Bipolar Disorder Tokyo Japan; ^3^ Division of Psychiatry, Tohoku Medical and Pharmaceutical University Sendai Japan

**Keywords:** bipolar disorder, consultation, peer group, psychotherapy, telephone consultation

## Abstract

**Aim:**

Previous research shows that telephone consultation is useful in suicide prevention, substance use disorder, and other mental illnesses. However, no study has been conducted with a specific focus on telephone consultation for patients with bipolar disorder (BPD). Therefore, this study investigates the utilization of telephone consultation by patients with BPD and their families and analyzes the consultation contents to identify specific issues that they face.

**Methods:**

We investigated a record book of telephone consultation conducted between 2013 and 2019 provided by the Japanese Alliance of Bipolar Disorder, which is a self‐help group in Japan specializing in BPD. The main themes regarding consultation were extracted and labelled as diagnosis, symptoms, treatment, laws and social support, interpersonal relations, social life, other people with BPD, and others, with up to three items being labeled per consultation.

**Results:**

A total of 3540 consultations were sought, and consulters who were patients accounted for 74% of the calls. The largest number of consultations were those related to symptoms (1522), followed by interpersonal relations (1003), social life (896), and treatment (797). There was a significant difference in the distribution of consultation contents between patients and their families (*χ* ^2^ = 44.595, *p* < 0.0001).

**Conclusion:**

Most consultations by patients with BPD were about their psychiatric symptoms. Consultation contents differed between patients and families, with patients focusing more on their own social life and families focusing more on the treatment of BPD. These findings could help health‐care professionals in formulating effective psychoeducation and psychotherapy programs.

## Graphical abstract

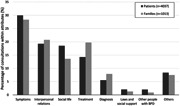

## INTRODUCTION

Telephone consultation is an important tool for providing mental health care. It helped reduce suicides rates and improved mental health conditions among young people in Australia.^1^ A literature review highlighted the positive effects of telephone consultation in treating drug or alcohol abuse.^2^, ^3^ An Indian study on the use of health‐care services provided via mobile phones for patients with severe mental diseases reported on the need for and benefits of crisis helplines.^4^ In recent years, due to the COVID‐19 pandemic, in which face‐to‐face interactions have been restricted, the use of mental health helplines has drawn attention worldwide.^5^, ^6^, ^7^, ^8^, ^9^ Additionally, the degree of dependence on crisis management helpline services has increased during lockdown periods due to increased isolation, aggravation of mental illnesses attributable to the pandemic, and limited access to or exclusion from other support resources.^8^ In Japan, telephone consultation is mostly used in cases pertaining to suicide prevention.^10^, ^11^ Moreover, it is used for consultation on perinatal care^12^ and childrearing,^13^ consultation with families of individuals who have withdrawn from society,^14^ and post‐traumatic stress disorder induced by earthquake disasters.^15^

One study on the users of telephone consultation examined the mental health profile of individuals calling one of the largest helpline services in Australia.^16^ However, this study was conducted on the general population, and no research was found specific to patients with bipolar disorder (BPD).

In addition to telephone consultation, another service is utilized in a similar manner via the Internet. In recent years, Internet‐based self‐help forums for patients with mental illness and their families have also become important receptacles for their concerns.^17^ However, it has been reported that the more frequently people use helplines, the more likely they are to lack access to the Internet,^16^ suggesting that the need for telephone‐based services remains high.

A study of online self‐help forums for BPD revealed that patients with BPD and their relatives seek acceptance and respect by communicating with other patients with BPD and their relatives in diagnosis‐specific self‐help forums, due to the lack of public understanding of the illness and the barriers to social interaction they face in “real life.”^18^ Similarly, telephone consultation by self‐help groups for BPD may represent needs different to those of the general population.

Patients with BPD often experience difficulties in areas of interpersonal relations, family, education, or employment, and have a deteriorating quality of life.^19^, ^20^, ^21^, ^22^ These difficulties persist even when the patient is in remission.^23^ If patients with BPD are diagnosed at an early stage of onset, their functional decline may be minimal; however, for approximately 30% of patients, there is a time gap of 10 or more years between the onset of symptoms and an accurate diagnosis and treatment.^19^, ^20^, ^24^, ^25^ Although the time taken to receive a diagnosis of BPD is becoming shorter in Argentina,^26^ in Japan, it takes about 4–8 years.^22^, ^27^ Faced with these circumstances, approximately 6%–7% of patients with BPD commit suicide, with their suicide rates being 20–30 times greater than those of the general population.^28^ Moreover, a questionnaire survey revealed that “having someone to consult with” was the principal factor that encouraged patients with BPD to live positively.^22^

Thus, telephone consultation can be useful for patients with BPD because of the difficulties they face in receiving a timely diagnosis, the resultant delay in treatment, and their problems in interpersonal relations and employment; moreover, patients could find comfort in consulting with counselors who are willing to listen. However, to the best of our knowledge, there are no studies on telephone consultation that focus specifically on patients with BPD. Therefore, this study aims to investigate the consultation contents of telephone consultation by patients with BPD and their families, and analyzes the consultation contents to identify specific issues that they face.

## METHODS

In this study, a record book of telephone consultation conducted between 2013 and 2019 was used. It was archived at the Japanese Alliance of Bipolar Disorder (JABD; http://bipolar-disorder.or.jp/), a self‐help group headquartered in Tokyo that arranges meetings, provides telephone consultation, issues bulletins, and so on^29^; it has 20 chapters across Japan. During this time, 715 days of telephone consultation were made available. As a basic rule, telephone consultation services were provided to patients with BPD who were members of JABD and their families. However, consultation requests by non‐members were also accepted. Professionals, including clinical psychologists, industrial counselors/therapists, peer counselors, and psychiatrists, offered telephone consultation. Prior to providing telephone consultation, peer counselors underwent 1 month of training at JABD.

From the record book of telephone consultation, the following data were extracted: date of consultation; residence address (name of the prefecture); consulter's age, sex, and relationship to the patient; patient's age and sex; and the main themes discussed during the consultation. As more than half of the consulters did not respond to the age question, it was excluded from analysis. Two experts in the field of mental health extracted the main themes of consultation and labeled them with instruction and supervision from a psychiatrist who was among the individuals that handled the telephone consultations. At first, the experts jointly labeled 602 cases, and after reaching a consensus on the label, they divided the remaining into two unequal parts. Expert A labeled 1276 cases and Expert B labeled 1662 cases, and they both crosschecked the labeling among themselves. Finally, the psychiatrist reviewed the labeling to ensure its correctness. The main themes regarding consultation were classified into diagnosis, symptoms, treatment, laws and social support, interpersonal relations, social life, other people with BPD, and others.

To compare the consultations of patients with the consultations of families, *χ* ^2^ tests with Bonferroni correction were performed. All statistical analyses were performed using EZR.^30^

Since this study covered past records, including many anonymous users, consent from individuals who had used telephone consultation services had not been obtained. Information on the implementation of research and contact information of the people in charge is published on the Tohoku Medical and Pharmaceutical University Hospital and the JABD websites. Additionally, the patients or their legal representatives (where applicable) were offered an opportunity to withdraw their participation from the study. This study was conducted after obtaining approval from the Clinical Research Review Board at Tohoku Medical and Pharmaceutical University Hospital (research registration No. 2021‐2‐082).

## RESULTS

Table 1 summarizes the information on the consultations and consulters' attributes. A total of 3540 telephone consultations (an average of five consultations per day) were sought, 2625 (74%) of which were from patients with BPD. In terms of sex, 2336 (66%) consulters were women, 1048 (30%) were men, and 156 (4%) were of unknown sex. Of the consulters who were patients, 1603 were women (61%), 901 were men (34%), and 121 (5%) were of unknown sex. The consulters belonged to different parts of Japan, although the addresses of many were unknown. Japanese nationals living in China and Australia also used the telephone consultation services.

**Table 1:** Information on telephone consultations and consulters' attributes

Variable	*n*	%
Total consultations	3540	
Days made available for consultations	715	
Average consultations per day	5.0	
Consulters by sex		
Female	2336	66.0
Male	1048	29.6
Unknown	156	4.4
Relationship to the patients		
Himself/herself	2625	74.2
Family	802	22.7
Others	43	1.2
Unknown	70	2.0
Consulters (patients) by sex		
Female	1603	61.1
Male	901	34.3
Unknown	121	4.6

*Note*: The period covered is 7 years, 2013–2019.

**Figure 1:**
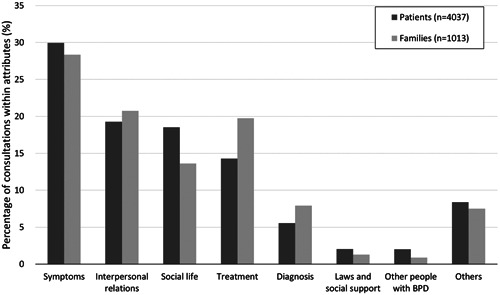
Themes regarding consultation. Black bars indicate consultation from patients with BPD. Of the 2625 consultations, up to three items were labeled per consultation, depending on the main themes. A total of 4037 items were labeled. Grey bars indicate consultations from families of patients with BPD. Of the 802 consultations, up to three items were labeled per consultation, depending on the main themes. A total of 1013 items were labeled. Consultations from persons whose relationship to the patient is unknown are excluded here.

Figure 1 shows the classification of the main themes regarding consultation in patients and their families. There was a significant difference in the topics discussed between patients and their families (*χ* ^2^ = 44.595, *p* = 0.0000001639). Multiple comparison tests with Bonferroni correction showed significant differences between “social life” and “treatment” (*p* < 0.0001), “social life” and “diagnosis” (*p* < 0.01), “symptoms” and “treatment” (*p* < 0.05), and “other people with BPD” and “treatment” (*p* < 0.05). In all the comparisons listed above, the former had a significantly higher percentage of patient consultations than family consultations.

The details of each label are described below for consultations from all sources, including not only patients and families, but also the persons who did not name whether they were the patient or a family.

### Symptoms

Of the 1522 consultations from the consulters that were classified under “symptoms,” the largest number of consultations—991 (65%)—were about depressive and manic states. Of these, regarding depressive states, the patients complained the most about depressive moods; for instance, several of them reported, “I am so depressed that I want to die” and “I want you to listen to how distressing my depression is.” Regarding manic states, many patients complained about the pain of not being able to control their mania. Specifically, they said, “I end up becoming irritable” and “I cannot manage my finances.” Besides these, many patients complained about experiencing both mania and depression and the agony of having mixed symptoms. Complaints other than those related to symptoms of BPD were often associated with sleep, such as “I do not feel sleepy,” “I feel terrible due to lack of sleep,” and “I end up waking up every 2–3 hours.” Moreover, several patients inquired about confronting their disease and controlling their symptoms; for instance, they said, “I cannot tell whether this is a symptom or a part of my personality,” “I do not know how to change my current state,” and “I was told that I might go into remission but will never be completely cured.”

### Interpersonal relations

Of the 1003 consultations that were classified under interpersonal relations, 734 (73%) were about problems with family relations. Specifically, the consulters complained, “My family does not understand me,” “I feel that I do not belong at home,” “My family is getting ruined because of me,” and so on. Moreover, problems related to interpersonal relations with people other than the family included, “I got hurt because of my relations with supporters,” “I do not know how to deal with people who are not family members,” “I tend to be distrustful in relationships with other people,” “My relations with other people are not going well,” and “I have lost numerous friends.”

### Social life

A total of 896 consultations regarding social life were received. They mainly pertained to work, school, reasons to live, and things that were worth doing. Of these, 695 (78%) were concerned with the patient's work. The patients who were on a leave of absence reported, “I am causing trouble for my company. Would it be better if I quit?,” “I have been taking so many leaves of absence that I feel sorry for my company,” and “Will I be able to resume work?” The patients who wanted to work often complained, “I do not know how to job hunt,” “I went for job interviews but failed each time,” “I cannot stay motivated to continue looking for work,” and “I cannot set goals successfully.” Several patients who were employed said, “Having revealed my illness, I feel increasingly uncomfortable at my workplace” and “After people learned about my illness, they stopped giving me interesting or worthwhile work.” Meanwhile, some patients posed positive, forward‐looking questions, such as “To fulfill my goal of returning to work, what should I pay attention to, in terms of managing my health?”

Regarding school, many consultations pertained to patients' anxiety about taking a leave of absence from school and their future plans. Peer staff members served as role models for patients who sought reasons to live and worked towards identifying things that were worth doing. Some comments appreciating the inspirational work of the peer staff read: “I have been able to view things more positively after learning that there are people like the peer staff members,” “I want to be like the peer staff members,” “I wrote an essay, so I want the peer staff members to read it,” (in the course of talking with a peer staff member who has certification as a nursing care helper) “I have decided to study to get certified as a nursing care helper myself,” and “I was able to set a goal for myself.”

### Treatment

Under the treatment label, there were 797 consultations. Of these, 418 (52%) were related to drug therapy. The following concerns were raised: “I am having lithium that was prescribed to me but do not get periodic blood tests done,” “They keep increasing the dose of my drugs,” “I had antidepressants prescribed, but is it safe to take them? Some information indicates that people with BPD should not take them,” “I am taking drugs faithfully as prescribed, but they are not working,” “I am anxious about taking drugs,” “I did not get the drug I want prescribed,” and “I suffer from intense side‐effects (e.g., trembling hands, headache, overproduction of saliva, and discomfort in my head).”

Many consultations concerned medical institutions and doctors. Specific comments included, “I want information on finding hospitals,” “I want to change my hospital,” and “Should we admit them to a hospital?” Several consultations about doctors showed the consulters' mistrust of and dissatisfaction with their primary doctors, such as “I do not know how to convey my feelings and symptoms to my primary doctor,” “I am not getting any better, so I do not trust my primary doctor,” “My primary doctor switches drugs without explaining them to me,” “My primary doctor does not listen to what I say,” and “My primary doctor would not write a medical certificate for me.”

### Diagnosis

Of the 308 consultations regarding diagnosis, 115 (37%) were about “accuracy of diagnosis.” Specifically, the patients complained, “I was diagnosed with depression for 8 years and have been taking antidepressants. Why could not the doctors detect that I had BPD sooner?” and “I wasted 10 years due to a misdiagnosis.” Other people reported, “After I switched hospitals, I was told I had a different disease,” “The diagnosis changes according to the psychiatrist's specialization,” and “I received this diagnosis unexpectedly, so there is no way I can accept it.”

### Laws and social support

There were 99 consultations under this label. A total of 57 (58%) consultations were related to disability pensions and 37 (37%) to driver's licenses, such as “What must I do to become eligible for a disability pension?,” “My disability pension was cut off,” “I could not update my driver's license” (in Japan, individuals with certain mental disorders, such as BPD, cannot update their driver's license without a medical certificate from a doctor), and “I did not declare my BPD when updating my driver's license. Have I broken the law?”

### Other people with BPD

There were 90 consultations under the label of “Other people with BPD.” Some patients wanted to know how other people with BPD were doing (54 consultations; 60%). Moreover, they complained about not having people with the same illness around them and wished to interact with such people. Comments such as the following were reported: “When conditions worsen, how does everyone else overcome them?,” “I want to connect with other people who have BPD like me,” and “I want to ask people with BPD about how they manage their everyday lives.”

### Others

A total of 467 consultations were sought under the label of “Others.” There were consultations about JABD, complaints of genetic concerns, current events, and trivial daily topics; 288 (62%) of the consultations were related to JABD. These included “What does JABD do?,” “How can I participate in meetings?,” and “Can you hold meetings closer to where I live?” The remaining 38% included comments such as “I'm worried about passing it on to my children and grandchildren.”

## DISCUSSION

According to our review of existing literature, this study is the first to analyze the content of telephone consultations for BPD patients and their families. Our results suggest that BPD patients and their families seek consultations for “symptoms,” “interpersonal relationships,” “social life,” and “treatment,” in that order of importance. In a previous study that analyzed the topics of the online self‐help forums for BPD patients, the topics discussed were “social networks,” “symptoms of illness,” and “treatment,” in descending order of importance.^18^ As “interpersonal relationships” and “social life” observed in this study are likely to be similar to “social networks” observed in the aforementioned study, we considered these two studies to have similar results, with BPD patients and their families primarily presenting concern regarding interpersonal relationships and social life. Despite the difference in format (telephone consultation vs. online forum), both studies targeted the activities of self‐help groups for BPD, who similarly represent the concerns of patients with BPD.

The distribution of consultations in the present study is somewhat different from that reported by Burgess et al.^16^ In the concerned study, nearly half of the respondents reported interpersonal concerns (51.2%), family problems (40.6%), and depression (46.5%) regardless of call frequency. However, when similar calculations were made using the survey data for this study, the results indicated concerns regarding family problems (20.7%), depression (28.0%), and nonfamily interpersonal relationships (7.6%). The telephone consultations in this study were limited to BPD, whereas those in the aforementioned study were targeted at the general population, which indicates the possibility of more consultations in this study being unique to BPD (e.g., treatment, diagnosis, and social life with BPD). This difference may also be due to the method used in our survey, which was to label up to three major issues in each consultation, whereas the previous study involved participants indicating the presence of each individual issue.

There were some significant differences in the distribution of consultations between patients and their families. This was mainly due to the ratio of “Social life” to “Treatment” being reversed (Figure 1). For the patients, their social life, including work, was identified to be more important than the treatment itself, while their families were more concerned about the patients' treatment. There are previous studies of questionnaire surveys that show that patients with BPD have higher levels of aggression when compared to patients experiencing depression and healthy individuals.^31^, ^32^ In the present study, families of patients with BPD expressed frequent concerns about verbal abuse and violence in their relationship with the patients. This may be part of the reason families are more concerned about the treatment than the social life of the patient. On the other hand, the patients have a greater focus on their social life in order to find the accurate balance to adjust in the society despite having BPD.

In this study, the largest number of consultations from both patients and their families were those pertaining to symptoms. It is understandable given the nature of BPD. Most patients with BPD go through periods of symptom and remission, with symptoms occurring about half the time during the course of the illness.^33^, ^34^ For this reason, it is believed that concerns about symptoms are serious and recurring, and consultations have become particularly frequent. In fact, it was observed that most patients called because they wanted someone to listen to their story and share the agony of their mental health concerns. Some of them inquired about specific methods for controlling their symptoms and mood swings. This might indicate the importance of having someone to talk to and consult with regarding the symptoms for patients with BPD. The immediacy of telephone consultation, that is, being able to seek immediate help when an individual feels distressed,^35^ may be especially effective in dealing with a mental illness such as BPD that is accompanied with emotional fluctuations. Patients can learn to deal with their symptoms and mood swings by effectively using telephone consultation and interacting closely with specialists and peer supporters, which may improve their overall mental state.^1^

In a past questionnaire survey for patients with BPD, about the same percentage of respondents cited interpersonal relations with “family” (46%) and “people other than family” (42%) as being aggravated because of their illness.^22^ The reason for the high percentage of consultations about family among the interpersonal relationships (over 70%) in the present results may be that BPD patients who have interpersonal problems with their families find it difficult to consult with them and rely on telephone consultation to seek support and help. It has been reported that inappropriate responses by family members can push patients over the edge,^29^ and it has been pointed out that BPD patients with family members with high expressed emotion in particular are at higher risk of BPD relapse.^36^

The present study also showed that work is particularly important in the social life of patients with BPD. In a past questionnaire survey, the long‐term inability to work was perceived as the greatest burden by patients with BPD.^22^ In another study, based on the Productivity and Activity Impairment Questionnaire items, patients with BPD who were employed had more than twice the level of absenteeism, presenteeism, and activity impairments than healthy controls.^37^ Several other studies have reported the severity of depressive episodes—rather than mania or hypomania—to be more related to loss of work productivity.^38^, ^39^, ^40^ Therefore, there might be a need to attend to patients' recurring anxieties, especially during their leave of absence to allow them to have a social role and a meaningful life. In this study, peer supporters served as role models for the consulters.

Regarding treatment, most consultations pertained to the effects and side‐effects of prescribed drugs. There were also many consultations about insufficient communication with the primary psychiatrist, suggesting that primary psychiatrists might not be providing sufficient explanations to the patients about the effects and side‐effects of prescribed drugs. In a past questionnaire survey, “better drug prescriptions” was the second most frequently cited service that patients sought from their primary psychiatrists.^41^

In terms of diagnosis, the patients who had been diagnosed with depression for a long time were particularly stressed by the change in diagnosis and treatment. When a diagnosis is changed, physicians need to be considerate and should help their patients accept and adapt to the change. Previous studies suggest that the longer the period from the onset of BPD to receiving a diagnosis and corresponding treatment, the more likely a patient experiences difficulties with interpersonal relations, family, education, or employment with a deteriorating impact in their quality of life.^19^, ^20^, ^21^, ^22^, ^24^, ^25^ However, making a prompt and accurate diagnosis of BPD remains challenging.

Of the consultations pertaining to laws and social support, approximately 56% and 39% were concerned with disability pensions and driver's licenses, respectively. The revision of Japan's Road Traffic Act (Article 33‐2‐3‐3‐1 of the Order for Enforcement of the Road Traffic Act; https://elaws.e-gov.go.jp/document?lawid=335CO0000000270_20210628_503CO0000000172), which was implemented in 2014, may have led to the large number of consultations related to updating driver's licenses. According to this act, patients with BPD were banned from obtaining a driver's license in case they “manifested symptoms that carried the risk of degrading their capabilities relating to cognition, estimation, judgment, or the physical ability necessary to drive a vehicle, etc., safely.” As a result, depending on the medical certificate of a patient, it became impossible for them to update their driver's license. Therefore, several patients contacted JABD to address their worries regarding this amendment in the law.

Regarding “Other people with BPD,” most consultations were about seeking out other people and their knowledge in the same circumstances. It appears that, in living with BPD, patients seek comrades with whom they can talk freely about their condition and learn from those who have been diagnosed before them about their coping mechanisms. Moreover, negative reactions from families, colleagues, and friends can aggravate the prognosis of patients^29^; therefore, forming relations with other patients, who are more likely to accept their condition, may provide considerable mental support.

As for the “Others” category, 62% of the respondents asked for specific advice about the contents of JABD. It is thought that these are the voices of patients with BPD and their families seeking knowledge and information based on actual experiences accumulated in peer groups. The remaining 38% included daily news topics, trivial daily reports, and, in a few cases, concerns about heredity. It is thought that there is a need to talk with someone even if there are no difficulties or symptoms to be consulted to seek comfort and to feel heard and understood.

Consultations were received from all over Japan. Telephone consultation allows people to seek help as per their convenience, irrespective of where they live.^35^ However, since the telephone consultation process cannot begin unless a person makes a phone call, it is important to publicize this service among affected individuals.^15^ It is believed that the activities carried out by JABD, such as publicity activities using the Web and SNS and sharing detailed schedules through bulletins, may have popularized telephone consultation.

Patients with BPD may use online forums of self‐help groups to address their social and emotional needs, such as feeling a sense of belonging and receiving the friendship and sympathy provided by the online group members.^18^ The telephone consultation that was the subject of this study was also run by a self‐help group. The needs of patients with BPD that are difficult to communicate to families and society may be concentrated in the activities of such self‐help groups.

However, this study has a few limitations. This study analyzed consultation contents from a single patient group in Japan; thus, the results cannot be generalized to patients with BPD worldwide. The diagnosis was provided by the patients and their families, and may not be considered reliable in some cases. As a certain number of users repeatedly use telephone consultation, their responses may have influenced the results of this study. For example, frequent callers are more likely to complain of anxiety, loneliness, and physical illness,^16^ which may have influenced the high number of consultations concerning symptoms observed.

## CONCLUSION

This paper analyzes the contents of telephone consultations by self‐help groups for BPD and investigates the most pressing issues faced by patients with BPD. The main topics discussed by patients with BPD during the telephone consultations were symptoms, followed by interpersonal relationships, social relationships, and treatment. The distribution of consultations differed between patients and family members, with patients featuring an increased focus on their own social life, and families featuring an increased focus on treatment for BPD. It is possible that the activities of self‐help groups condense the needs of patients with BPD in ways that are difficult to communicate to family and society. Additionally, we hope that the patient‐specific issues identified in this study will influence treatment and contribute to the development of psychoeducational programs and social policies. Despite the limitations mentioned above, we plan to continue to conduct similar surveys and carefully observe whether the needs of BPD patients change from year to year.

## AUTHOR CONTRIBUTIONS

Eiji Suzuki and Yuko Isogaya designed the study, collected, analyzed, and interpreted the data, and prepared and edited the manuscript. Chiho Suzuki collected the data and edited the manuscript. Shingo Hoshina and Masashi Nibuya analyzed and interpreted the data and prepared and edited the manuscript. All authors approved the final manuscript as submitted.

## CONFLICT OF INTEREST

The authors declare no conflict of interest.

## ETHICS APPROVAL STATEMENT

This study was conducted after obtaining approval from the Clinical Research Review Board at Tohoku Medical and Pharmaceutical University Hospital (research Registration No. 2021‐2‐082).

## PATIENT CONSENT STATEMENT

This study covered past records, including many anonymous users; consent from individuals who had used telephone consultation services had not been obtained. Information on the implementation of research and contact information of the people in charge is published on the Tohoku Medical and Pharmaceutical University Hospital and the JABD websites. Additionally, the patients or their legal representatives (where applicable) were offered an opportunity to withdraw their participation from the study.

## Data Availability

The data that support the findings of this study are available from the corresponding author, E.S., upon reasonable request.
